# 1,4-Bis(1*H*-benzimidazol-1-yl)but-2-ene

**DOI:** 10.1107/S1600536811024251

**Published:** 2011-06-25

**Authors:** Gui-Ying Dong, Tong-Fei Liu, Cui-Huan Jiao, Xiao-Chen Deng, Xiao-Ge Shi

**Affiliations:** a College of Chemical Engineering, Hebei United University, Tangshan 063009, People’s Republic of China; b Qian’an College, Hebei United University, Tangshan 063009, People’s Republic of China

## Abstract

In the pseudo-centrosymmetric mol­ecule of the title compound, C_18_H_16_N_4_, two benzimidazole fragments form the dihedral angles of 83.49 (7) and 79.37 (7)°, with the mean plane of the linking butene chain. No classical inter­molecular inter­actions are observed. The porous crystal packing exhibits voids of 85 Å^3^.

## Related literature

For applications of benzimidazole derivatives, see: Tidwell *et al.* (1993 ▶); Santra & Dogra (1999 ▶). For related structures, see: Su *et al.* (2003 ▶); Chen *et al.* (2007 ▶); Liu *et al.* (2011 ▶).
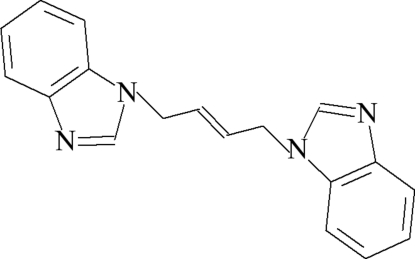


## Experimental

### 

#### Crystal data

C_18_H_16_N_4_
*M* *_r_* = 288.35
Monoclinic, 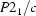

*a* = 12.564 (3) Å
*b* = 9.140 (2) Å
*c* = 18.131 (3) Å
β = 127.281 (12)°
*V* = 1656.7 (6) Å^3^
*Z* = 4
Mo *K*α radiation
μ = 0.07 mm^−1^
*T* = 295 K
0.20 × 0.18 × 0.17 mm

#### Data collection

Bruker SMART CCD area-detector diffractometer
Absorption correction: multi-scan (*SADABS*; Sheldrick, 1996 ▶) *T*_min_ = 0.956, *T*_max_ = 0.996
12226 measured reflections
2925 independent reflections
1639 reflections with *I* > 2σ(*I*)
*R*_int_ = 0.056

#### Refinement

*R*[*F* ^2^ > 2σ(*F* ^2^)] = 0.052
*wR*(*F* ^2^) = 0.133
*S* = 1.01
2925 reflections
199 parameters
H-atom parameters constrained
Δρ_max_ = 0.19 e Å^−3^
Δρ_min_ = −0.14 e Å^−3^

### 

Data collection: *SMART* (Bruker, 1998 ▶); cell refinement: *SAINT* (Bruker, 1998 ▶); data reduction: *SAINT*; program(s) used to solve structure: *SHELXS97* (Sheldrick, 2008 ▶); program(s) used to refine structure: *SHELXL97* (Sheldrick, 2008 ▶); molecular graphics: *SHELXTL* (Sheldrick, 2008 ▶); software used to prepare material for publication: *SHELXTL* and *PLATON* (Spek, 2009 ▶).

## Supplementary Material

Crystal structure: contains datablock(s) I, global. DOI: 10.1107/S1600536811024251/cv5116sup1.cif

Structure factors: contains datablock(s) I. DOI: 10.1107/S1600536811024251/cv5116Isup2.hkl

Supplementary material file. DOI: 10.1107/S1600536811024251/cv5116Isup3.cml

Additional supplementary materials:  crystallographic information; 3D view; checkCIF report

## Figures and Tables

**Fig. 1.**
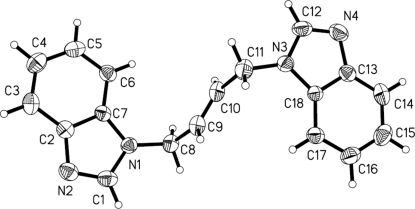
The molecular structure of (I), showing displacement ellipsoids at the 30% probability level.

## supplementary crystallographic information

### Comment

Bis-benzimidazole compounds have have been widely used due to their anti-viral activities (Tidwell *et al.*, 1993), photochemical and photophysical properties (Santra & Dogra, 1999). They have found applications in supramolecular coordination chemistry to generate various coordination architectures (Su *et al.*, 2003; Chen *et al.*, 2007; Liu *et al.*, 2011). Herewith we report the crystal structure of the title bis-benzimidazole compound (I).

In (I) (Fig. 1), the molecule adopts a *trans* conformation. Two benzimidazole fragments form the dihedral angles of 83.49 (7) and 79.37 (7) °, respectively, with the mean plane of linking them butene chain. The C11—C10—C9—C8 torsion angle is 176.5 (3)°. The average bond distances and angles for the benzimidazole ring are in agreement with those in related benzimidazole compounds (Chen *et al.*, 2007; Liu *et al.*2011). In the absence of classical intermolecular interactions, the porous crystal packing exhibits voids of 85 Å^3^.

### Experimental

The title compound was prepared according to the literature (Liu *et al.*, 2011). Single crystals were grown from an ethanol solution over a period of several days at room temperature.

### Refinement

H atoms were placed in calculated positions (C—H  0.93-0.97 Å), and refined with a riding model, with *U*_iso_(H) = 1.2*U*_eq_(C). The program Squeeze in *PLATON* (Spek, 2009) was applied to remove regions of diffuse electron density that could not be satisfactorily modeled.

### Crystal data

**Table:**

C_18_H_16_N_4_	*F*(000) = 608
*M**_r_* = 288.35	*D*_x_ = 1.156 Mg m^−^^3^
Monoclinic, *P*2_1_/*c*	Mo *K*α radiation, λ = 0.71073 Å
Hall symbol: -P 2ybc	Cell parameters from 4528 reflections
*a* = 12.564 (3) Å	θ = 3.5–20.3°
*b* = 9.140 (2) Å	µ = 0.07 mm^−^^1^
*c* = 18.131 (3) Å	*T* = 295 K
β = 127.281 (12)°	Block, colourless
*V* = 1656.7 (6) Å^3^	0.20 × 0.18 × 0.17 mm
*Z* = 4	

### Data collection

**Table:**

Bruker SMART CCD area-detector diffractometer	2925 independent reflections
Radiation source: fine–focus sealed tube	1639 reflections with *I* > 2σ(*I*)
graphite	*R*_int_ = 0.056
φ and ω scans	θ_max_ = 25.0°, θ_min_ = 2.0°
Absorption correction: multi-scan (*SADABS*; Sheldrick, 1996)	*h* = −14→14
*T*_min_ = 0.956, *T*_max_ = 0.996	*k* = −10→10
12226 measured reflections	*l* = −21→21

### Refinement

**Table:**

Refinement on *F*^2^	Primary atom site location: structure-invariant direct methods
Least-squares matrix: full	Secondary atom site location: difference Fourier map
*R*[*F*^2^ > 2σ(*F*^2^)] = 0.052	Hydrogen site location: inferred from neighbouring sites
*wR*(*F*^2^) = 0.133	H-atom parameters constrained
*S* = 1.01	*w* = 1/[σ^2^(*F*_o_^2^) + (0.0545*P*)^2^] where *P* = (*F*_o_^2^ + 2*F*_c_^2^)/3
2925 reflections	(Δ/σ)_max_ < 0.001
199 parameters	Δρ_max_ = 0.19 e Å^−^^3^
0 restraints	Δρ_min_ = −0.14 e Å^−^^3^

### Special details

**Table:**

Geometry. All e.s.d.'s (except the e.s.d. in the dihedral angle between two l.s. planes) are estimated using the full covariance matrix. The cell e.s.d.'s are taken into account individually in the estimation of e.s.d.'s in distances, angles and torsion angles; correlations between e.s.d.'s in cell parameters are only used when they are defined by crystal symmetry. An approximate (isotropic) treatment of cell e.s.d.'s is used for estimating e.s.d.'s involving l.s. planes.
Refinement. Refinement of *F*^2^ against ALL reflections. The weighted *R*-factor *wR* and goodness of fit *S* are based on *F*^2^, conventional *R*-factors *R* are based on *F*, with *F* set to zero for negative *F*^2^. The threshold expression of *F*^2^ > σ(*F*^2^) is used only for calculating *R*-factors(gt) *etc*. and is not relevant to the choice of reflections for refinement. *R*-factors based on *F*^2^ are statistically about twice as large as those based on *F*, and *R*- factors based on ALL data will be even larger.

### Fractional atomic coordinates and isotropic or equivalent isotropic displacement parameters (Å^2^)

**Table:**

	*x*	*y*	*z*	*U*_iso_*/*U*_eq_	
N1	0.93808 (17)	−0.12271 (19)	0.16716 (12)	0.0514 (5)	
N2	1.0262 (2)	−0.1093 (2)	0.31752 (13)	0.0646 (6)	
N3	0.55655 (17)	0.1198 (2)	−0.20779 (12)	0.0533 (5)	
N4	0.4402 (2)	0.1102 (2)	−0.36219 (14)	0.0724 (6)	
C1	1.0390 (2)	−0.0742 (2)	0.25325 (18)	0.0632 (7)	
H1	1.1111	−0.0206	0.2655	0.076*	
C2	0.9075 (2)	−0.1874 (2)	0.27030 (15)	0.0528 (6)	
C3	0.8415 (3)	−0.2500 (3)	0.30264 (17)	0.0650 (7)	
H3	0.8766	−0.2436	0.3648	0.078*	
C4	0.7230 (3)	−0.3217 (3)	0.2396 (2)	0.0710 (7)	
H4	0.6771	−0.3641	0.2595	0.085*	
C5	0.6706 (2)	−0.3319 (3)	0.14720 (18)	0.0662 (7)	
H5	0.5907	−0.3822	0.1068	0.079*	
C6	0.7322 (2)	−0.2708 (2)	0.11317 (16)	0.0567 (6)	
H6	0.6962	−0.2778	0.0509	0.068*	
C7	0.8516 (2)	−0.1974 (2)	0.17686 (14)	0.0474 (6)	
C8	0.9207 (2)	−0.0957 (2)	0.08116 (15)	0.0592 (6)	
H8A	0.8973	−0.1865	0.0469	0.071*	
H8B	1.0043	−0.0612	0.0955	0.071*	
C9	0.8136 (2)	0.0152 (3)	0.02248 (17)	0.0590 (6)	
H9	0.8277	0.1096	0.0460	0.071*	
C10	0.7016 (2)	−0.0108 (2)	−0.05950 (17)	0.0600 (6)	
H10	0.6902	−0.1037	−0.0843	0.072*	
C11	0.5908 (2)	0.0964 (3)	−0.11649 (16)	0.0678 (7)	
H11B	0.5122	0.0616	−0.1235	0.081*	
H11A	0.6166	0.1892	−0.0839	0.081*	
C12	0.4456 (2)	0.0739 (3)	−0.2902 (2)	0.0684 (7)	
H12	0.3783	0.0207	−0.2953	0.082*	
C13	0.5584 (2)	0.1877 (2)	−0.32404 (15)	0.0548 (6)	
C14	0.6068 (3)	0.2551 (3)	−0.36690 (17)	0.0738 (8)	
H14	0.5599	0.2508	−0.4308	0.089*	
C15	0.7263 (3)	0.3282 (3)	−0.3115 (2)	0.0797 (8)	
H15	0.7597	0.3761	−0.3388	0.096*	
C16	0.7985 (2)	0.3327 (3)	−0.2160 (2)	0.0699 (7)	
H16	0.8794	0.3827	−0.1807	0.084*	
C17	0.7535 (2)	0.2653 (2)	−0.17234 (16)	0.0545 (6)	
H17	0.8021	0.2673	−0.1082	0.065*	
C18	0.6318 (2)	0.1941 (2)	−0.22858 (14)	0.0443 (5)	

### Atomic displacement parameters (Å^2^)

**Table:**

	*U*^11^	*U*^22^	*U*^33^	*U*^12^	*U*^13^	*U*^23^
N1	0.0494 (11)	0.0484 (11)	0.0549 (12)	−0.0002 (9)	0.0308 (11)	0.0018 (9)
N2	0.0630 (13)	0.0578 (13)	0.0591 (13)	−0.0018 (11)	0.0297 (11)	−0.0044 (10)
N3	0.0467 (11)	0.0536 (12)	0.0573 (12)	0.0023 (10)	0.0303 (10)	0.0052 (10)
N4	0.0592 (14)	0.0724 (15)	0.0568 (13)	−0.0074 (12)	0.0201 (11)	−0.0030 (11)
C1	0.0531 (15)	0.0533 (16)	0.0684 (18)	−0.0049 (12)	0.0291 (15)	−0.0048 (13)
C2	0.0570 (15)	0.0439 (13)	0.0556 (15)	0.0049 (12)	0.0331 (13)	−0.0007 (11)
C3	0.084 (2)	0.0591 (16)	0.0631 (16)	0.0103 (14)	0.0507 (16)	0.0061 (12)
C4	0.0794 (19)	0.0583 (17)	0.094 (2)	0.0030 (15)	0.0625 (18)	0.0055 (15)
C5	0.0515 (15)	0.0570 (16)	0.084 (2)	−0.0026 (13)	0.0376 (15)	0.0007 (13)
C6	0.0537 (15)	0.0529 (15)	0.0532 (14)	0.0025 (12)	0.0269 (13)	0.0030 (11)
C7	0.0455 (13)	0.0417 (13)	0.0488 (14)	0.0046 (11)	0.0253 (12)	0.0022 (10)
C8	0.0641 (15)	0.0595 (15)	0.0607 (15)	0.0039 (13)	0.0413 (13)	0.0064 (12)
C9	0.0732 (16)	0.0531 (15)	0.0619 (16)	0.0067 (13)	0.0468 (15)	0.0067 (12)
C10	0.0753 (17)	0.0540 (15)	0.0641 (16)	0.0089 (14)	0.0492 (15)	0.0122 (12)
C11	0.0686 (17)	0.0706 (17)	0.0766 (18)	0.0148 (14)	0.0504 (15)	0.0169 (13)
C12	0.0478 (15)	0.0543 (16)	0.082 (2)	−0.0080 (12)	0.0285 (16)	0.0017 (14)
C13	0.0522 (15)	0.0512 (14)	0.0527 (14)	0.0033 (12)	0.0274 (13)	−0.0001 (11)
C14	0.083 (2)	0.082 (2)	0.0539 (16)	0.0163 (16)	0.0402 (16)	0.0155 (14)
C15	0.080 (2)	0.079 (2)	0.105 (2)	0.0119 (17)	0.0691 (19)	0.0235 (17)
C16	0.0555 (15)	0.0593 (17)	0.092 (2)	−0.0005 (13)	0.0430 (16)	0.0075 (14)
C17	0.0485 (14)	0.0457 (14)	0.0588 (15)	0.0036 (11)	0.0271 (13)	0.0026 (11)
C18	0.0420 (13)	0.0384 (12)	0.0490 (14)	0.0039 (11)	0.0258 (11)	0.0027 (10)

### Geometric parameters (Å, °)

**Table:**

N1—C1	1.358 (3)	C8—C9	1.495 (3)
N1—C7	1.382 (2)	C8—H8A	0.9700
N1—C8	1.458 (2)	C8—H8B	0.9700
N2—C1	1.309 (3)	C9—C10	1.308 (3)
N2—C2	1.387 (3)	C9—H9	0.9300
N3—C12	1.352 (3)	C10—C11	1.491 (3)
N3—C18	1.389 (2)	C10—H10	0.9300
N3—C11	1.452 (3)	C11—H11B	0.9700
N4—C12	1.308 (3)	C11—H11A	0.9700
N4—C13	1.393 (3)	C12—H12	0.9300
C1—H1	0.9300	C13—C18	1.384 (3)
C2—C7	1.389 (3)	C13—C14	1.387 (3)
C2—C3	1.395 (3)	C14—C15	1.372 (3)
C3—C4	1.375 (3)	C14—H14	0.9300
C3—H3	0.9300	C15—C16	1.387 (3)
C4—C5	1.383 (3)	C15—H15	0.9300
C4—H4	0.9300	C16—C17	1.365 (3)
C5—C6	1.368 (3)	C16—H16	0.9300
C5—H5	0.9300	C17—C18	1.383 (3)
C6—C7	1.391 (3)	C17—H17	0.9300
C6—H6	0.9300		
			
C1—N1—C7	105.92 (18)	H8A—C8—H8B	108.0
C1—N1—C8	127.2 (2)	C10—C9—C8	124.8 (2)
C7—N1—C8	126.83 (18)	C10—C9—H9	117.6
C1—N2—C2	104.1 (2)	C8—C9—H9	117.6
C12—N3—C18	105.52 (19)	C9—C10—C11	125.3 (2)
C12—N3—C11	127.5 (2)	C9—C10—H10	117.3
C18—N3—C11	126.95 (19)	C11—C10—H10	117.3
C12—N4—C13	103.8 (2)	N3—C11—C10	113.18 (19)
N2—C1—N1	114.2 (2)	N3—C11—H11B	108.9
N2—C1—H1	122.9	C10—C11—H11B	108.9
N1—C1—H1	122.9	N3—C11—H11A	108.9
N2—C2—C7	110.3 (2)	C10—C11—H11A	108.9
N2—C2—C3	130.0 (2)	H11B—C11—H11A	107.8
C7—C2—C3	119.7 (2)	N4—C12—N3	114.8 (2)
C4—C3—C2	117.8 (2)	N4—C12—H12	122.6
C4—C3—H3	121.1	N3—C12—H12	122.6
C2—C3—H3	121.1	C18—C13—C14	119.7 (2)
C3—C4—C5	121.3 (2)	C18—C13—N4	110.2 (2)
C3—C4—H4	119.3	C14—C13—N4	130.1 (2)
C5—C4—H4	119.3	C15—C14—C13	117.6 (2)
C6—C5—C4	122.3 (2)	C15—C14—H14	121.2
C6—C5—H5	118.8	C13—C14—H14	121.2
C4—C5—H5	118.8	C14—C15—C16	121.7 (2)
C5—C6—C7	116.3 (2)	C14—C15—H15	119.1
C5—C6—H6	121.9	C16—C15—H15	119.1
C7—C6—H6	121.9	C17—C16—C15	121.6 (2)
N1—C7—C2	105.48 (19)	C17—C16—H16	119.2
N1—C7—C6	131.9 (2)	C15—C16—H16	119.2
C2—C7—C6	122.6 (2)	C16—C17—C18	116.5 (2)
N1—C8—C9	111.31 (18)	C16—C17—H17	121.8
N1—C8—H8A	109.4	C18—C17—H17	121.8
C9—C8—H8A	109.4	C17—C18—C13	122.9 (2)
N1—C8—H8B	109.4	C17—C18—N3	131.3 (2)
C9—C8—H8B	109.4	C13—C18—N3	105.72 (19)
			
C2—N2—C1—N1	0.1 (3)	C12—N3—C11—C10	108.2 (3)
C7—N1—C1—N2	−0.3 (2)	C18—N3—C11—C10	−71.6 (3)
C8—N1—C1—N2	176.94 (19)	C9—C10—C11—N3	123.4 (2)
C1—N2—C2—C7	0.2 (2)	C13—N4—C12—N3	−0.2 (3)
C1—N2—C2—C3	−178.6 (2)	C18—N3—C12—N4	−0.1 (3)
N2—C2—C3—C4	179.6 (2)	C11—N3—C12—N4	−179.9 (2)
C7—C2—C3—C4	0.8 (3)	C12—N4—C13—C18	0.4 (2)
C2—C3—C4—C5	0.2 (3)	C12—N4—C13—C14	−178.9 (2)
C3—C4—C5—C6	−0.7 (4)	C18—C13—C14—C15	−1.1 (3)
C4—C5—C6—C7	0.2 (3)	N4—C13—C14—C15	178.1 (2)
C1—N1—C7—C2	0.4 (2)	C13—C14—C15—C16	1.4 (4)
C8—N1—C7—C2	−176.86 (18)	C14—C15—C16—C17	−0.5 (4)
C1—N1—C7—C6	−179.6 (2)	C15—C16—C17—C18	−0.7 (3)
C8—N1—C7—C6	3.1 (3)	C16—C17—C18—C13	1.0 (3)
N2—C2—C7—N1	−0.4 (2)	C16—C17—C18—N3	−177.8 (2)
C3—C2—C7—N1	178.58 (19)	C14—C13—C18—C17	−0.1 (3)
N2—C2—C7—C6	179.66 (19)	N4—C13—C18—C17	−179.48 (19)
C3—C2—C7—C6	−1.3 (3)	C14—C13—C18—N3	178.9 (2)
C5—C6—C7—N1	−179.1 (2)	N4—C13—C18—N3	−0.4 (2)
C5—C6—C7—C2	0.8 (3)	C12—N3—C18—C17	179.2 (2)
C1—N1—C8—C9	−104.7 (2)	C11—N3—C18—C17	−0.9 (3)
C7—N1—C8—C9	72.0 (3)	C12—N3—C18—C13	0.3 (2)
N1—C8—C9—C10	−116.2 (2)	C11—N3—C18—C13	−179.85 (19)
C8—C9—C10—C11	176.37 (19)		

## References

[bb1] Bruker (1998). *SMART* and *SAINT* Bruker AXS Inc., Madison, Wisconsin, USA.

[bb2] Chen, C.-L., Zhang, J.-Y. & Su, C.-Y. (2007). *Eur. J. Inorg. Chem.* pp. 2997– 3010.

[bb3] Liu, T.-F., Wu, W.-F., Wan, C.-Q., He, C.-H., Jiao, C.-H. & Cui, G.-H. (2011). *J. Coord. Chem.* **64**, 975–986.

[bb4] Santra, S. & Dogra, S. K. (1999). *J. Mol. Struct.* **476**, 223–233.

[bb5] Sheldrick, G. M. (1996). *SADABS* University of Göttingen, Germany.

[bb6] Sheldrick, G. M. (2008). *Acta Cryst.* A**64**, 112–122. 10.1107/S0108767307043930 18156677

[bb7] Spek, A. L. (2009). *Acta Cryst.* D**65**, 148–155. 10.1107/S090744490804362X PMC2631630 19171970

[bb8] Su, C.-Y., Cai, Y.-P., Chen, C.-L., Smith, M. D., Kaim, W. & zur Loye, H.-C. (2003). *J. Am. Chem. Soc.* **125**, 8595–8613. 10.1021/ja034267k 12848568

[bb9] Tidwell, R. R., Jones, S. K., Naiman, N. A., Berger, L. C., Brake, W. B., Dykstra, C. C. & Hall, J. E. (1993). *Antimicrob. Agents Chemother.* **37**, 1713–1716. 10.1128/aac.37.8.1713 PMC188050 8215291

